# Benchmark dose calculations for PFAS exposure based on two data sets on immunotoxic effects

**DOI:** 10.1186/s12940-023-00985-w

**Published:** 2023-05-06

**Authors:** Esben Budtz-Jørgensen, Philippe Grandjean

**Affiliations:** 1 Section of Biostatistics, Department of Public Health, University of Copenhagen, Øster Farimagsgade 5, DK-1014 Copenhagen, Denmark; 2 Department of Environmental Medicine, University of Southern Denmark, Odense, Denmark; 3 Department of Environmental Health, Harvard School of Public Health, MA 02215 Boston, USA; 4 Department of Pharmacology and Biomedical Sciences, University of Rhode Island, Kingston RI 02881, USA

**Keywords:** PFAS exposure, Immune function, Benchmark dose, Risk assessment

## Abstract

**Background:**

Exposure to perfluorinated alkylate substances (PFAS) is associated with harmful effects on human health, including developmental immunotoxicity. This outcome was chosen as the critical effect by the European Food Safety Authority (EFSA), which calculated a new joint reference dose for four PFAS using a Benchmark Dose (BMD) analysis of a study of 1-year old children. However, the U.S. Environmental Protection Agency (EPA) recently proposed much lower exposure limits.

**Methods:**

We explored the BMD methodology for summary and individual data and compared the results with and without grouping for two data sets available. We compared the performance of different dose-response models including a hockey-stick model and a piecewise linear model. We considered different ways of testing the assumption of equal weight-based toxicity of the four PFAS and evaluated more flexible models with exposure indices allowing for differences in toxicity.

**Results:**

Results relying on full and decile-based data were in good accordance. However, BMD results for the larger study were lower than observed by EFSA for the smaller study. EFSA derived a lower confidence limit for the BMD of 17.5 ng/mL for the sum of serum-PFAS concentration, while similar calculations in the larger cohort yielded values of about 1.5 ng/mL. As the assumption of equal weight-based toxicity of the four PFAS seems questionable, we confirmed dose-dependencies that allowed potency differences between PFAS. We also found that models linear in the parameters for the BMD analysis showed superior coverage probabilities. In particular, we found the piecewise linear model to be useful for Benchmark analysis.

**Conclusions:**

Both data sets considered could be analyzed on a decile basis without important bias or loss of power. The larger study showed substantially lower BMD results, both for individual PFAS and for joint exposures. Overall, EFSA’s proposed tolerable exposure limit appears too high, while the EPA proposal is in better accordance with the results.

## Background

In the absence of feasible means to determine thresholds for toxic effects in humans, calculation of the so-called Benchmark dose (BMD) has been proposed as a well-defined mathematical solution to obtaining an appropriate point of departure [1, 2]. This approach has been adopted by regulatory agencies as a routine procedure [3–5]. As a starting point, one has to determine the magnitude of a change that should be considered disadvantageous; for example, an increase of 5% in a serum parameter or a single IQ point [3, 5] may be considered adverse. The dose measure that is associated with this change is called the BMD, and its lower one-sided 95% confidence limit is called the Benchmark dose level (BMDL). Such calculations have already been applied to a range of environmental chemicals, most recently the perfluorinated alkylate substances (PFASs) [6].

In the final risk assessment from 2020 [6], the European Food Safety Authority decided that immunotoxicity in children should be regarded as the critical effect, i.e., an adverse outcome that occurs at the lowest exposure levels, as determined by PFAS concentrations in blood. This decision relied on several prospective studies in children and in adults [7–9]. However, BMD calculations from observational data where exposures are not determined by design and unexposed controls are absent, represent a challenge. While the lack unexposed subjects can be handled by extrapolation [7], a Benchmark analysis of observational data also need to adjust for a variety of covariables to allow for possible differences in background profiles between study participants [10]. Regulatory agencies are confronted by an additional limitation, because data may have to be censured, e.g., for reasons of legal protection of personal data. This hurdle had to be considered in the recent EFSA approach to BMDL calculations [6].

In order to rely on a single study, EFSA chose a recently published study of 101 children from Germany [11], where individual data could be scanned from a published graph. We compare with findings from a larger Faroese cohort study [7, 12], where we have access to the full data base. Both studies measured the concentrations of specific antibodies against routine childhood vaccines and documented lower antibody responses in children with elevated PFAS exposures, as documented by analyses of concomitant blood samples and/or cord blood or maternal pregnancy blood. At the request from EFSA, we provided detailed BMD calculations as well as access to decile-based data. However, in the absence of the full data from the Faroes study, EFSA decided to rely on their own BMD calculations that were based on the German study [11].

In mid-2022, the U.S. Environmental Protection Agency announced a substantial decrease of the so-called Reference Dose (RfD) for PFOS and PFOA and therefore also the guideline for drinking water contamination [13]. For PFOA, the draft RfD is 1.5 pg/kg $\cdot$ day, and for PFOS 7.9 pg/kg $\cdot$ day [13]. These advisories are about 100-fold lower than the joint EFSA tolerable weekly intake (TWI) of 4.4 ng/kg $\cdot$ week [14]. Although details have not yet been released, the basis for this change is the BMDL results obtained from our study of vaccine antibody responses to routine childhood vaccinations [15].

Based on the analysis of the data from the Faro Islands and Germany on the health effects of PFAS exposure, the present study explores methods for benchmark calculations in human data. Both in 2017 and 2022, EFSA have published guidelines on the BMD approach and both reports concluded that specific guidance for the analysis of human data is needed [4, 16]. However, such a document still does not exist and we hope that the results of the present paper will help improve general guidelines of Regulatory Agencies. We therefore first examine the biostatistical approach to BMD calculations when either full or decile-based data are available. We compare the performance of different dose-response models including the linear model, a hockey-stick model and a piecewise linear model, that are generally not used in benchmark analysis of experimental data. In the EFSA’s BMD analysis, the total concentration of the four most prevalent PFASs in serum was used as the exposure indicator [17]. This decision assumes that the weight-based potency of the four PFASs is the same, therefore we also consider methods for Benchmark analysis based on an exposure index which does not assume equal potency.

## Methods

In accordance with the EFSA recommendations [6], we calculated BMD results for the sum of the four PFAS (PFOA, PFOS, PFHxS and PFNA) concentrations in serum using first the (individual level) data, then the decile version of the data provided to EFSA, while comparing results from the Faroese and the German studies. For the individual level Faroese data, calculations were based on regression models with antibody concentrations as dependent continuous variables while serum-PFAS concentrations were included as independent variables along with potential confounders as previously reported [7, 12]. To achieve normally distributed residuals, antibody concentrations were log-transformed. Thus, models were on the following form:

1 $$\begin{aligned} \log Y (d, z_1, \ldots , z_p) = \alpha _0 +\sum \limits _{j=1}^p \alpha _j z_j+f(d) + \epsilon , \end{aligned}$$

where $Y(d, z_1, \ldots , z_p)$ is the antibody concentration for a subject, *d* is the sum of PFAS concentrations (PFOA, PFOS, PFHxS and PFNA) and $z_1, \ldots z_p$ are covariate values. The dose-response function (*f*) satisfies $f(0)=0$. We modeled the PFAS effect using a linear-dose response function $[f(d)=\beta d],$ and because the relationship at low doses may differ from the one at higher doses, we also used a piecewise linear model [$f(d)=\beta _1 (d 1_{d<d_0} + d_0 1_{d>d_0})+ \beta _2 (d-d_0) 1_{d>d_0}]$, which allowed for a difference in slopes below and above the median exposure level ($d_0$). An important advantage of these two models is that they are linear in the parameters, so that the parameter estimates have well-defined statistical properties that can be utilized to derive lower confidence limits for the BMD with coverage probabilities close to the nominal level. This is important for BMD analysis as the main result (the so-called BMDL) is given as a one-sided lower 95% confidence limit for the BMD.

**Fig. 1:**
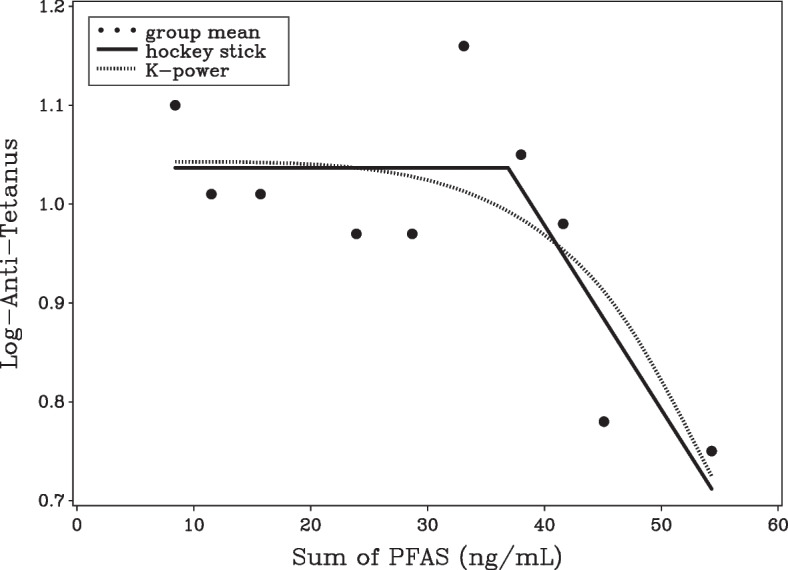
Scatter plot of association between the sum of serum-PFAS concentrations (ng/mL) and log-transformed tetanus antibody concentration from the decile data of the German study [11]. The fully drawn curve was estimated from the hockey stick model, while the dotted curve was estimated in the *K*-power model

Sometimes a close fit to the data cannot be achieved with linear models, and more flexible models must be considered. For example, the German data showed a weak slope in the low dose range for both diphtheria and tetanus antibodies. This led the authors to use a so-called hockey stick model $[f(d)=\beta (d-\gamma )1_{d>\gamma }]$, where the slope is assumed to be zero up to an unknown threshold ($\gamma$) which is estimated from the data [18]. Above the threshold, a linear slope is assumed. The EFSA report [6] presents results from the hockey stick model for the sum PFAS concentration. In this model, the dose level corresponding to a reduction of one standard deviation in the antibody concentrations was estimated. Although such calculations are useful, statistical uncertainty needs to be taken into account. Thus, the Benchmark methodology is extended to the hockey stick model, while estimating the parameters using the R-package *segmented* [19]. Unfortunately, the hockey stick model is known for poor statistical properties [20] and therefore we also included the (restricted) *K*-power model $[f(d)=\beta d^K, \, K\ge 1]$, which is well established for Benchmark analysis [21] and also able to produce a fit with a near-zero slope up to an unknown exposure level followed by a more pronounced effect (see Fig. 1).

### BMD estimation and calculation of the BMDL

The BMD was defined as the dose leading to a predefined reduction in the antibody concentration. This reduction is denoted as the BMR, and is often chosen to be 5% or 10%. Given that the outcome (*Y*) is log-normally distributed it is natural to define the BMD as the dose associated with a pre-specified reduction in the geometric mean, i.e., as the solution in *d* to the following equation

2 $$\begin{aligned} \frac{\text {geometric mean}[Y(d, z_1, \ldots , z_k)]}{ \text {geometric mean}[Y(0,z_1, \ldots , z_k )]}=1-\text {BMR}. \end{aligned}$$

with $\text {geometric mean}[Y(d, z_1, \ldots , z_k)]= \exp (E[\log Y(d, z_1, \ldots , z_k)])$. Using a logarithmic transformation on both sides of the equation we get

3 $$\begin{aligned} E[\log Y(d, z_1, \ldots , z_k)]-E[\log Y(0,z_1, \ldots , z_k )]=\log (1-\text {BMR}). \end{aligned}$$

In general models, the solution will depend on covariates ($z_1,\ldots , z_p$) which is not practical for risk assessment. However, under the additive model (1), the BMD will be constant across covariate values and its value is given as the solution (in *d*) to the following equation

4 $$\begin{aligned} f(d) = \log (1-\text {BMR}) \end{aligned}$$

For example, in the linear model $\text {BMD}=\log (1-\text {BMR})/\beta$ if $\beta <0$, while in the hockey stick model $\text {BMD}= \gamma +\log (1-\text {BMR})/\beta$, if $\beta <0$. Appendix A provides BMD expressions for the piecewise linear model. Based on a given data set, the BMD is obtained by first estimating the parameters of *f* and then plugging them into the BMD expression. If parameters are estimated consistently, this procedure will lead to consistent BMD estimations.

The lower confidence limit, the BMDL, can be calculated in various ways. The BMD is typically a non-linear function of model parameters and therefore its estimate does not follow a normal distribution, meaning that standard Wald confidence limits will not have good properties. Instead the profile likelihood based limits are generally recommended [21, 22]. If we let $L(\theta )$ denote the likelihood function in a model with parameter vector $\theta$, then the profile likelihood function for BMD is obtained after re-parametrization so that the BMD becomes one of the model parameters $\theta =(\tilde{\theta }, \text {BMD})$. The profile likelihood function for the BMD is $L_{p}(\text {BMD})=max_{\tilde{\theta }} L(\tilde{\theta }, \text {BMD})$, and the lower one-sided 95% confidence limit is

5 $$\begin{aligned} \text {BMDL} = \min [\text {BMD}: 2\{\log [L(\widehat{\theta })]-\log [L_p(\text {BMD})]\} \le \chi ^2_1(90)], \end{aligned}$$

where $\chi ^2_1(90) \approx 2.71$ is the 90’th percentile in the $\chi ^2$-distribution with one degree of freedom.

In correctly specified models that are linear in the parameters (e.g. linear and piecewise linear model), the distribution of the regression coefficients describing *f*(*d*) is known to be normal and therefore a closed form BMDL expression can be determined without relying on asymptotic results. First, a lower confidence limit [*LC*(*d*)] for *f*(*d*) is determined and then the BMDL is obtained as the solution to

6 $$\begin{aligned} LC(d)=\log (1-\text {BMR}) \end{aligned}$$

For example, in the linear model $LC(d)=[\widehat{\beta }-t(df,95) s.e.(\widehat{\beta })] d$ and $\text {BMDL}=\log (1-\text {BMR})/[\widehat{\beta }-t(df,95) s.e.(\widehat{\beta })]$ if $\widehat{\beta }-t(df,95) s.e.(\widehat{\beta })]<0$, where $\widehat{\beta }$ is the estimated value of $\beta$ with standard error $s.e.(\widehat{\beta })$ and *t*(*df*, 95) is the 95’th percentile in the *t*-distribution with *df* degrees of freedom. In addition to being simple to calculate, this solution is exact meaning that the coverage probability will be 95% no matter the sample size. In Appendix A, we use the same procedure for deriving a BMDL expression in the piecewise linear model.

For the hockey stick model, we calculate the BMDL using Bootstrap re-sampling as results from standard asymptotic likelihood theory are unlikely to be appropriate given the limited sample size available here. By definition, decile data hold information from only ten different exposure levels. With the non-parametric Bootstrap, we would get re-sampled data sets with even fewer different exposure levels. Thus, we instead used the parametric Bootstrap. For each outcome, we generated 1000 data sets by simulating from the conditional outcome distribution given covariates from the original data. In each data set, the BMD is estimated, and the BMDL is obtained as the 5th percentile in the distribution of BMD estimates.

### Evaluation of models: fit and BMDL coverage

An important practical challenge in BMD analysis is that different models may yield different BMDLs. When evaluating the results, one must consider how well the given model fits the data, but the statistical properties of the corresponding BMDL should also be taken into account. Thus, a complex model may have a relatively good fit, but its Benchmark results may not be reliable if the BMDL has a coverage probability far from the nominal value.

The model fit was based on minus two times the log-maximum likelihood function [$-2\log (L)$], and the Akaike Information Criterion (AIC) of $-2\log (L)+2p$, where *p* is the number of parameters in the model. For both measures, a smaller value indicates a better fit, but the AIC is often used by regulatory agencies as the fit of larger models is penalized and because this measure allows comparisons of non-nested models. Coverage probabilities were explored in simulations studies. For each model, parameters were first estimated using the original data and then the 1000 data sets were generated from the model using the estimated parameter values from the original data. In each of 1000 data sets, we calculated the BMDL, and the coverage probability was estimated as the proportion of data sets where the BMDL was higher than the BMD. Thus, in these simulations we examine the performance of each BMDL in situations where the model is correctly specified.

### Benchmark analysis based on summary data

This section briefly discusses statistical consequences of using summary data instead of the individual data when conducting Benchmark analysis of continuous outcome data. We first consider a linear dose-response model

7 $$\begin{aligned} Y_i=\alpha + \beta d_i +\epsilon _i, \end{aligned}$$

where $Y_i$ is the response and $d_i$ is exposure concentration of subject *i*. The residual term $\epsilon _i$ is assumed to follow a normal distribution $\epsilon _i \sim N(0,\sigma ^2)$.

We consider data with exposure groups $g=1,...,K$ (e.g. decile groups). In each group, we have the mean exposure $\overline{d}_g$ and the mean outcome $\overline{Y}_g$. From Eq. 7 it is easy to derive the distribution of the average outcome

8 $$\begin{aligned} \overline{Y}_g=\alpha + \beta \overline{d}_g +\epsilon _g, \end{aligned}$$

where $\epsilon _g \sim N(0,\sigma ^2/n_g)$ and $n_g$ is the sample size in group *g*. Therefore we conclude that parameters $\alpha$ and $\beta$ can be estimated without bias using the summary data. However, estimates from the summary data will have a larger variance. This means that if the Benchmark dose is defined as a function of the parameters $\alpha$ and $\beta$ then a consistent estimate of the BMD can be obtained from the summary data, but because of the increased estimation uncertainty BMDLs will tend to be lower compared to those from the individual level data.

In non-linear models, analysis based on summary data will generally not be unbiased. For illustration we consider the model $Y_i=\alpha + \beta f(d_i) +\epsilon _i$, where *f* is a known non-linear dose-response function. Here $\beta$ could be estimated with no bias using the covariate $\overline{f(d)}_g$ - the mean transformed exposure in each group. However, this quantity cannot be obtained from the summary data. One can transform the mean exposure, but that will not be equal to the mean transformed exposure, i.e., $f(\overline{d}_g) \ne \overline{f(d)}_g$. This problem might be addressed by increasing the number of groups so that the dose response is approximately linear within each group to obtain $f(\overline{d}_g) \approx \overline{f(d)}_g$. However, for non-linear models, some degree of bias from using summary data must be expected even in large data sets. It is interesting that if groupings are chosen suitably then a piecewise linear model with specific break-points can be estimated consistently in summary data. This is the case for the German data [11] as it consists of decile data that include the mean exposure in each group. If the break-point is placed at the median (or one of the other deciles) then the model can be consistently estimated as the dose-response relation will be linear in all decile groups.

### Evaluation of the hypothesis of equal potency and estimation of alternative weights

Previous calculations were based on models on the form

9 $$\begin{aligned} \log Y (d_1,d_2, d_3,d_4, z_1, \ldots , z_p) = \alpha _0 + \sum \limits _{j=1}^p \alpha _j z_j + \beta \, \text {index} + \epsilon , \end{aligned}$$

where the exposure index is the sum of concentrations to $d_1=$PFOA, $d_2=$PFOS, $d_3=$PFHxS and $d_4=$PFNA. Such analysis relies on an assumption of equal potency. In this section, we evaluate the hypothesis of equal potency in the Faroese data, and develop more general exposure indices.

The hypothesis of equal weights was evaluated using a more flexible model including the four PFAS concentrations as covariates

10 $$\begin{aligned} \log Y (d_1,d_2, d_3,d_4, z_1, \ldots , z_p) = \alpha _0 +\sum \limits _{j=1}^p \alpha _j z_j + \sum \limits _{k=1}^4 \beta _k d_k + \epsilon , \end{aligned}$$

where $\text {d}_k, k=1,...,4$ denotes the four PFAS concentrations. In this model we tested the hypothesis of equal potency $H_0: \beta _1=\beta _2=\beta _3=\beta _4$ using a likelihood ratio test comparing models (9) and (10). Furthermore, we tested the appropriateness of EFSA’s index by adding each of the PFAS concentrations (PFOS, PFOA, PFHxS or PFNA) to model (9) as an additional covariate, i.e.,

11 $$\begin{aligned} \log Y (d_1,d_2, d_3,d_4,z_1\ldots , z_p) = \alpha _0 +\sum \limits _{j=1}^p \alpha _j z_j + \beta \, \text {index} + \beta _k d_k + \epsilon , \end{aligned}$$

where $k=1,...,4$. If the PFAS effect is closely approximated by the index variable, then we would expect to see only a weak effect of $d_k$ in this model. Therefore, in model (11), we tested the hypothesis $H_0: \beta _k=0$ and recorded the *p*-value.

Next, we explored the sensitivity of Benchmark results to the assumption of equal potency by using the Faroese data to estimate alternative weights. Thus, we considered more general exposure indices on the form

12 $$\begin{aligned} \text {index}=4 \, (w_1 \text {PFOA} + w_2 \text {PFOS} + w_3 \text {PFHxS} + w_4 \text {PFNA}), \end{aligned}$$

where the sum of the weights ($$w_1, w_2, w_3, w_4)$$ equals 1. The summed concentration is a special case, where are weights are equal. We estimated weights in an approach similar to weighted quantile sum (WQS) regression [23]. Thus, we linked the exposure index to the antibody outcome in a linear model

13 $$\begin{aligned} \log Y (d_1,d_2, d_3,d_4,z_1, \ldots , z_p) = \alpha _0 + \sum \limits _{j=1}^p \alpha _j z_j + \beta \,\sum \limits _{k=1}^4 4 w_k d_k + \epsilon , \end{aligned}$$

Here the parameter $\beta$ describes the effect of the index, while the weights describe the contribution of each PFAS. The model was fitted under the restriction $\sum _i w_i =1$ and $w_i \ge 0, i=1,\ldots ,4$. In practise, this can be done using a standard regression model

14 $$\begin{aligned} \log Y (d_1,d_2, d_3,d_4,z_1, \ldots , z_p) = \alpha _0 + \sum \limits _{j=1}^p \alpha _j z_j + \sum \limits _{k=1}^4 \theta _k d_k + \epsilon , \end{aligned}$$

where regression coefficients $\theta _k, k=1,...,4$ are restricted to be non-positive. In a given data, the weights can be estimated as $\widehat{w}_l=\widehat{\theta }_l/(\sum _k \widehat{\theta }_k)$. To achieve more stable estimation, the final weights of the different PFAS concentrations were estimated by generating 1000 versions of the data using Bootstrap sampling. In each sample, the model was fitted, and the weights were estimated. Finally, the weight was estimated as the mean across the Bootstrap data. Weights were estimated using the raw exposure concentrations and based on concentrations that had been standardized to achieve a mean of zero and a variance of 1. Having estimated the weights, we then calculated Benchmark results for the corresponding index using the linear and the piecewise linear model. The latter calculation did not take uncertainty of the weights into account and therefore the BMDLs may have been overestimated [24].

## Results

To help the overview, this section is divided into two parts: one part that is focused on the risk assessment for PFAS and one that is focused on the statistical properties of the proposed BMD methodology. The first part mainly compares results from the two studies and here we focus at a BMR of 10% as this value was selected by EFSA.

### Risk assessment for PFAS

In the Faroese cohort data, we saw a close agreement in Benchmark results for tetanus and diphtheria (Table 1). For both outcomes the power parameter *K* was estimated at 1 and the linear model provided a fit which was almost as good as the best fitting model (piecewise linear). In the linear model, BMDLs at a BMR of 10% were about 3.0 ng/mL, while the piecewise linear model showed a BMDL$_{10}$ of about 1.3 ng/mL. As expected, these results were in agreement with results obtained when data were grouped into decile groups (Table 2).

**Table 1:** Benchmark results for the age-5 serum sum of serum-PFAS concentrations (ng/mL) in regard to tetanus and diphtheria antibody concentrations at age 7 years in the Faroese individual data [15]. $-2\log (L)$ is minus two times the log-maximum likelihood function and *p* is the number of parameters. $\Delta$AIC is the difference in AIC [$-2\log (L)+2p$] of current model and the model with the minimum AIC (disregarding Hockey stick model as BMDL was not available). Coverage is the coverage probability of the BMDL as estimated in a simulation study

		BMR=5%		BMR=10%					
Antibody	DR-model	BMD	BMDL	BMD	BMDL	$-2 \log (L)$	*p*	$\Delta$AIC	coverage
Tetanus	Linear	2.577	1.434	5.294	2.946	1717.94	5	0	0.944
	*K*-power	2.577	1.448	5.294	2.959	1717.94	6	2.00	0.880
	Piecewise	1.516	0.673	3.113	1.383	1717.59	6	1.65	0.951
	Hockey stick	15.067	-	17.762	-	1717.91	6	-	-
Diphtheria	Linear	2.672	1.513	5.488	3.108	1656.10	5	0	0.954
	*K*-power	2.672	1.531	5.488	3.123	1656.10	6	2	0.890
	Piecewise	1.222	0.632	2.511	1.298	1655.02	6	0.92	0.951
	Hockey stick	2.672	-	5.488	-	1656.10	6	-	-

**Table 2:** Benchmark results for the age-5 serum sum of PFAS concentrations (ng/mL) in regard to tetanus and diphtheria antibody concentrations at age 7 years in decile data of the Faroese study [15]. $-2\log (L)$ is minus two times the log-maximum likelihood function and *p* is the number of parameters. $\Delta$AIC is the difference in AIC [$-2\log (L)+2p$] of current model and the model with the minimum AIC. Coverage is the coverage probability of the BMDL as estimated in a simulation study

		BMR=5%		BMR=10%					
Antibody	DR-model	BMD	BMDL	BMD	BMDL	$-2 \log (L)$	*p*	$\Delta$AIC	Coverage
Tetanus	Linear	2.110	1.290	4.334	2.649	-1.42	2	0	0.957
	*K*-power	2.110	1.373	4.334	2.815	-1.42	3	2.00	0.812
	Piecewise	1.965	0.683	4.037	1.404	-1.43	3	1.99	0.949
Diphtheria	Linear	2.073	1.203	4.259	2.471	1.52	2	0	0.952
	*K*-power	2.073	1.286	4.259	2.640	1.52	3	2.00	0.801
	Piecewise	1.243	0.531	2.553	1.091	1.01	3	1.49	0.944

In the German data set (Fig. 1), the association between antibodies against Haemophilus infuenza type B (HiB) and PFAS concentrations was well described using a linear model (Table 3). Thus, in the power model, *K* was estimated at 1 and the linear model had the best AIC-value. For this outcome a BMDL$_{10}$ of 2.5 ng/mL was obtained from the linear and *K*-power model, while the piecewise linear model yield a value of 1.5 ng/mL. For tetanus and diphtheria, the *K*-power model, the piecewice linear model and the hockey stick model fitted clearly better than the linear model. The fit showed a flat curve at low doses and a steep curve at elevated exposures (Fig. 1). This finding agrees with the hockey stick analysis presented in the EFSA report. As a consequence of the relatively weak slope at low doses, Benchmark results were higher than the HiB results, with BMDL$_{10}$-values ranging from about 7.8 - 17.1 ng/mL (piecewise linear model) to 19.8-24.2 ng/mL (*K*-power model). For these two outcomes, the full data Benchmark analysis has been published in the recent EFSA report. Despite the strong non-linear dose-response relationship, we see a reasonably good agreement with the decile-based results. Thus, the full data analysis with a BMR of 10% and using the *K*-power model yielded BMD$_{10}$=31.6 ng/mL, BMDL$_{10}$=17.6 ng/mL for diphtheria, while the corresponding result from our grouped analysis was BMD$_{10}$=35.8 ng/mL, BMDL$_{10}$=24.2 ng/mL. Full data results were not provided for HiB by EFSA as they found the exposure effect to be statistically insignificant in all models that were considered.

**Table 3:** Benchmark results for the sum of serum-PFAS concentrations (ng/mL) in regard to tetanus, diphtheria and HiB antibody concentrations in the decile data of the German study [11]. $-2\log (L)$ is minus two times the log-maximum likelihood function and *p* is the number of parameters. $\Delta$AIC is the difference in AIC [$-2\log (L)+2p$] of current model and the model with the minimum AIC (disregarding Hockey stick model as BMDL was not available). Coverage is the coverage probability of the BMDL as estimated in a simulation study

		BMR=5%		BMR=10%					
Antibody	DR-model	BMD	BMDL	BMD	BMDL	$-2 \log (L)$	*p*	$\Delta$AIC	Coverage
Tetanus	Linear	4.213	2.326	8.654	4.778	-18.90	2	2.54	0.949
	*K*-power	31.10	13.51	36.16	19.82	-23.18	3	0.26	0.918
	Piecewise	36.90	3.810	38.63	7.825	-23.44	3	0	0.954
	Hockey stick	38.08	-	39.34	-	-25.92	3	-	-
Diphtheria	Linear	3.575	1.864	7.343	3.829	-13.14	2	6.51	0.941
	*K*-power	31.29	18.64	35.78	24.22	-20.17	3	1.48	0.895
	Piecewise	40.96	8.309	42.14	17.07	-21.65	3	0	0.951
	Hockey stick	37.58	-	38.56	-	-22.27	3	-	-
HiB	Linear	2.107	1.148	4.327	2.357	-4.44	2	0	0.955
	*K*-power	2.107	1.236	4.327	2.533	-4.44	3	2.00	0.818
	Piecewise	2.075	0.724	4.261	1.487	-4.44	3	2.00	0.940
	Hockey stick	13.52	-	15.66	-	-4.55	3	-	-

When comparing the results form the two studies, we see a nice agreement between the Faroese results and the results for HiB in the German study, with BMDL$_{10}$-values of 2.5-3.0 ng/mL for the linear model and 1.3-1.5 ng/mL for the piecewise linear model. However, for tetanus and diphtheria the German results are clearly higher than the Faroese. Thus, for the *K*-power model the German BMD$_{10}$-values are approximately teen times higher than those from the Faro Islands study.

In the Faroese data, we evaluated the appropriateness of the EFSA index by testing the hypothesis of equal PFAS potency ($H_0: \beta _1=\beta _2=\beta _3=\beta _4$). The evidence against this hypothesis was not strong for diphtheria, but the likelihood ratio test was borderline significant for tetanus ($p=0.069$). In further investigations, we considered regression models which included the summed index together with one of the four PFAS concentrations. For tetanus, we found a significant ($p=0.020$) adverse effect of PFOA even after adjusting for the EFSA index. This indicates that the index does not sufficiently describe the effect of PFOA. More complex models allowing for different weights showed that PFOA was generally an important predictor, while PFHxS and PFNA had smaller weights. Despite these differences the Benchmark results were quite stable across the different indices.

### Benchmark methodology

In these data, models that are linear in the parameters (linear and piecewise linear) performed well. They fitted data (almost) as closely as more complex models and the BMDL had coverage probabilities very close to the nominal level also in data with a limited sample size.

**Fig. 2:**
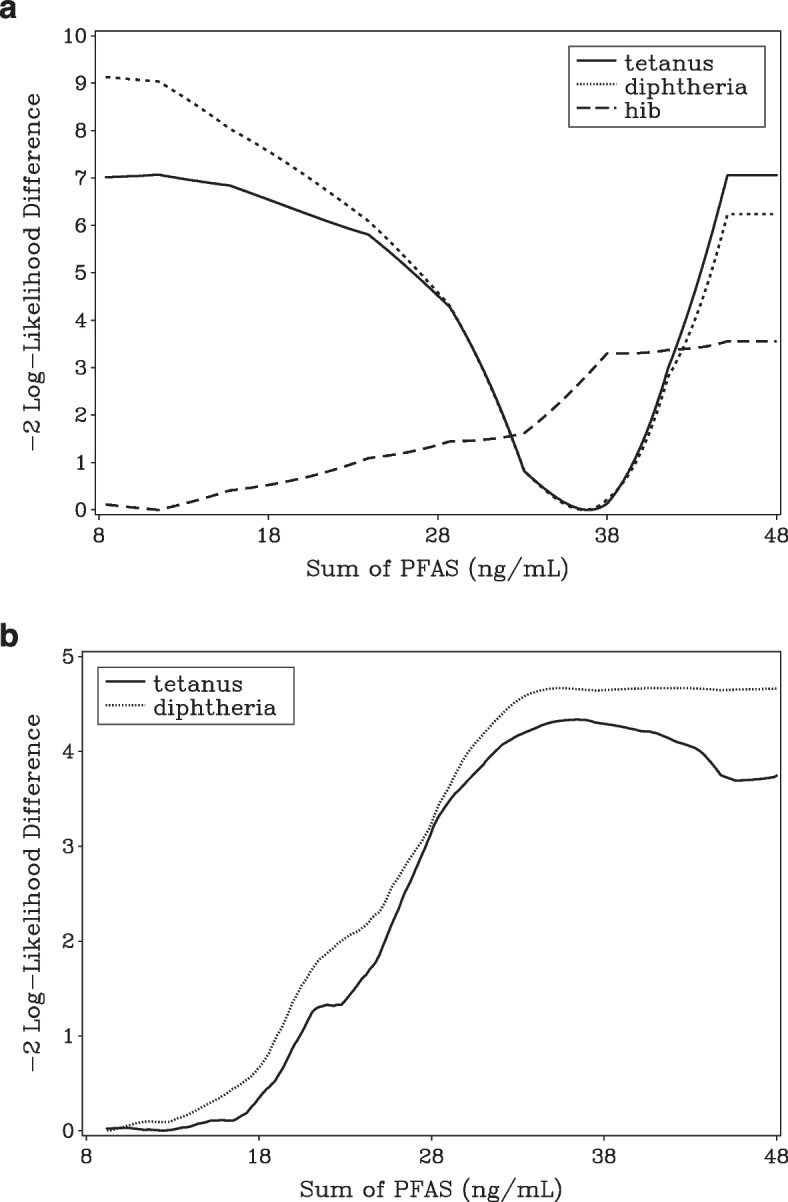
Profile likelihood function for the break-point in the hockey stick model. In the German study [11] (top graph), tetanus and diphtheria concentrations indicates a break-point around 37 ng/mL, while the HiB concentration and the Faroese data [15] (lower graph) show no signs of a break-point. The Faroese curves are both non-concave and for diphtheria the best fitting model is a linear curve with no break-point

While the threshold is fixed in the piecewise linear model, in the hockey stick model it is estimated from the data. This has important consequences for the performance of the model. As illustrated in Fig. 2, the likelihood function may not be concave and estimation techniques may get stuck in a local minimum and may not correctly identify the global minimum. In the Faroese data, there is very little information supporting the existence of a threshold. In fact for diphtheria, the global minimum of the profile likelihood function was achieved for thresholds below the lowest observed dose, meaning that a linear model without a threshold has the closest fit. However, the algorithm of the R-package “segmented” estimates a threshold at 12.9 ng/mL. This finding of course has important consequences for BMD estimation. The BMD estimate of the hockey stick model should have been 2.67 ng/mL, but instead the algorithm erroneously gives BMD$=$15.5 ng/mL. It may be possible to fine-tune the algorithm to achieve the correct solution in the current data, but the BMDL calculation would require correct calculation also in a high number of re-sampled Bootstrap data. The problem was less critical for tetanus and diphtheria in the German data both showing evidence of a threshold around 37 ng/mL (Fig. 1). This was correctly estimated with the “segmented” algorithm. However, because of the incorrect result in the Faroese data set, we are not convinced that reliable confidence limits are available and we cannot recommend this for general use in Benchmark calculations. Still, as shown in Fig. 2, the profile likelihood function of the break-point may be a useful supplement to scatter plots when exploring the possibility of a break-point in dose-response data.

As an alternative to the hockey stick model, we included the *K*-power model (see Fig. 1). This model produces smooth dose-response functions and is expected to have better statistical properties. However, also for this model, problems were observed with the coverage probabilities below the expected level. In fact, for none of the outcomes the coverage probabilities of the profile likelihood method was larger than 92%. There are two reasons for the poor performance: a limited sample size and inference on the boundary of the parameter space. For tetanus and diphtheria in the German data, the power parameter *K* was estimated to be far above 1, so the second problem is not relevant, but the decile sample size is low ($n=10$) and the BMDL depends on the asymptotic behavior of the likelihood function. Better results were obtained by instead using the (parametric) Bootstrap for BMDL calculation. This resulted in lower BMDLs for tetanus and diphtheria (6.52 ng/mL and 10.68 ng/mL) with coverage probabilities of 95.9% and 95.4%, respectively. In the Faroese data, the sample size is not small, but *K* was estimated at 1. When parameters are on the boundary of the parameter space, standard results from maximum likelihood theory may not hold [25, 26] and therefore the coverage probabilities are below 95%. Again we tried to use confidence intervals based on the Bootstrap, but this led to low coverage probabilities of around 80%. This may seem surprising, but also the Bootstrap method is known to be sensitive to parameters on the boundary [27].

Thus, we have seen that in data with small sample sizes, the Bootstrap approach out-preformed the profile likelihood with regard to coverage probabilities for BMDL calculation in the *K*-power model. However, when $\widehat{K} \approx 1$ the performance of the Bootstrap deteriorated, and it is unclear how to achieve appropriate confidence intervals using the *K*-power model. Since $K=1$ corresponds to the linear model, a simple solution would be to base the Benchmark analysis on the linear model only.

## Discussion

In our detailed Benchmark analyses of PFAS immunotoxicity we examined a variety of dose-response models in two different data sets with several outcome variables. It is clearly challenging to derive a reference dose level based on multiple BMDLs. Still, EFSA has provided guidelines of how to proceed in this situation [3, 4, 16]. At the time of this calculation, the recommended procedure was roughly speaking: for each outcome, results from all models that fit almost as a as well as the best fitting model is retained ($\Delta \text {AIC} \le 2$), and the BMDL for the outcome is given as the lowest of the retained BMDLs. Variability across outcomes is dealt with by choosing the minimum BMDL. The U.S.EPA has similar guidelines [5]. If we use this procedure for the Faroese data for the PFAS sum, we have to choose the piecewise linear model and a BMDL of around 1.5 ng/mL for a BMR of 10% (Table 1). If one instead considers the German decile data, a very similar BMDL is obtained based on the HiB concentration. These results are all based on the piecewise linear model which has so far not been applied by regulatory agencies. If this model is disregarded, there is still a close agreement between the two studies with a BMDL of about 3-4 ng/mL (Table 1).

EFSA had access to the scanned German data and to a decile version of the Faroese data. Based on the evidence that EFSA relied upon, a BMDL of 17.5 ng/mL was chosen for a BMR of 10% [6]. This result was obtained for the diphtheria concentration using the *K*-power model in the German data. There are two main reasons why EFSA’s BMDL is higher than the one we derived. First, EFSA decided not to use the Faroese results due to the absence of legal access to the individual data. Second, EFSA decided not to consider BMDLs from the HiB concentration in the German data. The choice not to consider HiB was in apparent agreement with EFSA’s standard procedure, because the association was not statistically significant in all models considered. However, a requirement of statistical significance in the Benchmark approach could be considered controversial and seems not to have been considered by the U.S.EPA (2012) and a requirement of statistical significance seems to have excluded in EFSA’s recently updated guideline [16]. The Benchmark approach is built on the fundamental idea that the risk assessment should be based on an upper confidence limit of risk. Restricting calculations to statistically significant effects at $p<0.05$ corresponds to redefining the conditions for the lower confidence band. This concern is especially relevant for observational human studies where it may not have been possible to reach a sample size providing an appropriate power.

This study explored a number of extensions of the current methodology for Benchmark analysis of observational data. First of all, we considered the performance of dose-response models that are currently not usually recommended by regulatory agencies. As the main result of a Benchmark analysis is the lower confidence limit (BMDL), we emphasize the importance of using models where confidence intervals have the correct coverage probability. As could be expected, models that are linear in the parameters show superior performance. Such models are often sufficiently flexible to allow modelling of human observational data, where exposure effects are often relatively weak compared to the residual variation. The piecewise linear model proved useful at least as a sensitivity analysis. Dose-response relations may differ at low and high exposures and the Benchmark calculation should not be affected the curve-shape at very high exposure concentrations. This is efficiently avoided in the piecewise linear model. We derived closed-form expressions for the BMD and the BMDL in this model and showed that the BMDL had coverage probabilities close to 95%. We recommend that these models will play a more important role in future guidelines for Benchmark analysis of human data. Past guidelines have not considered the piecewise linear model, while the linear model has been given little attention. Thus, this model was completely ignored in the recently updated EFSA guidelines, but the WHO mentioned it as a possible model for benchmark analysis in human data based on similar experiences as ours showing that often this model provides a sufficient fit to observational human data.

The possibility of using summary (e.g., decile-based) data for Benchmark analysis was also considered. In regard to experimental data, with information from a limited number of exposure groups, a reliable BMD analysis can be achieved from the summary data [28]. However, when grouping observational data, some individuals’ exposure level will differ from the level assigned to their group. Generally, this exposure mis-classification will lead to a bias in the estimation of the dose-response function and the BMD. However, if the dose-response is linear, then the grouped analysis will be unbiased but less powerful. As a result the BMDL may be biased, but in this case the bias will be towards lower and more safe dose levels. In the Faroese data, the dose-response was well approximated by a linear function and a good agreement was observed between the individual and the group-level BMD analysis. This advantage may not be present in non-linear relations, but a possible solution could be to increase the number of subgroups. WHOs recent guidelines on Benchmark analysis describes the possibility of analysis of human data based on summary statistics, but the potential bias from the induced exposure error is ignored [29].

In regard to the exposure index, EFSA’s approach was to use the arithmetic sum of serum concentrations of the four PFAS chosen represents the combined PFAS impact on the outcome. However, the molecular weights differ from a low of about 400 (PFHxS) to a high of 500 (PFOS). Accordingly, the same mass will contain rather more PFHxS than of PFOS, and the molar-based potency of PFHxS is therefore assigned to be 80% of the one for PFOS. Although this is no doubt a practical approach, there is little evidence to support this choice. An alternative could be to assign equal molar potencies, although again, little documentation is available. In fact, more than one mode of action may be in operation, and an approach to obtaining a joint measure of the total potency is not easily identified [30]. In our modeling approaches (Table 4), we used an exposure index that was defined by the data. It allowed consideration of the relative potency of the individual PFAS, as indicated by effects on antibody concentrations. Perhaps not surprisingly, PFOS and PFOA obtained the greatest weight, although their higher serum concentrations provided better exposure precision that may have supported a greater weight. The relative potencies obtained from these calculations differ substantially from the equal weight-based potencies assumed by EFSA and also from a uniform molar potency. Our results suggest that more attention need to be placed also on this aspect of Benchmark calculations for combined exposures. Alternatively, the idea of Benchmark analysis for a PFAS index could be abandoned and exposure limits calculated for each PFAS possibly after adjustment for other exposures. We have previously published such results [12] and the new EPA limits seems to have used this strategy.

**Table 4:** Estimated weights of different PFAS and Benchmark results for the corresponding exposure index. Analysis was restricted to antibody concentrations in Faroese individual data [15]. Indices were generated from the four serum-PFAS concentrations. Furthermore, weights were estimated using raw exposure concentrations (Raw) and after standardization (Stand.) of the concentrations. Benchmark results are for the age-5 serum weighted sum of PFAS serum concentrations (ng/mL)

		Weights					BMR=5%		BMR=10%	
Antibody	Method	PFOA	PFOS	PFHXS	PFNA	Model	BMD	BMDL	BMD	BMDL
Tetanus	Raw	0.739	0.059	0.163	0.040	linear	1.402	0.905	2.880	1.860
						piecewise	1.232	0.515	2.532	1.059
	Stand.	0.698	0.145	0.134	0.023	linear	1.761	1.115	3.618	2.290
						piecewise	1.494	0.629	3.069	1.292
Diph.	Raw	0.570	0.208	0.125	0.096	linear	2.488	1.467	5.111	3.014
						piecewise	1.336	0.667	2.745	1.370
	Stand.	0.456	0.408	0.085	0.052	linear	3.896	2.249	8.003	4.619
						piecewise	1.862	0.967	3.824	1.986

### Conclusions

Overall our results indicate that EFSA’s exposure limit [6] may be too high. Further, a joint limit for the four PFASs seems poorly justified, when based on the total weight. In comparison, EPA’s recently proposed limits for PFOS and PFOA [13] individually appear in much better accordance with our calculations [7, 12]. Because the details of EPA’s kinetic calculations are not yet publicly available, an exact comparison cannot be carried out at this time. Still, if EPA used a default 10-fold uncertainty factor, the difference from EFSA’s tolerable exposure limit can be explained by the lower BMDLs derived from the Faroese data.

## Data Availability

The dataset analyzed in this study is not publicly available due to national data security legislation on sensitive personal data.

## Appendix: Benchmark analysis in piecewise linear model

Here we derive expressions for BMD and BMDL in the piecewise linear model with a known break-point $d_0$. The model is

15 $$\begin{aligned} \log Y (d, z_1, \ldots , z_p) = \alpha _0 +\sum \limits _{j=1}^p \alpha _j z_j + \beta _1 x_1 + \beta _2 x_2 + \epsilon , \end{aligned}$$

with $x_1=d$ for $d<d_0$ and $x_1=d_0$ for $d>d_0$; and $x_2=0$ for $d<d_0$ and $x_2=d-d_0$ for $d>d_0$. So for doses below the break-point the slope is $\beta _1$, while the slope is $\beta _2$ above the break-point.

The Benchmark dose (BMD) is the dose *d* which leads to a decreased response level of BMR$\times 100\%$. Thus, the BMD is found by solving the following equation in *d*.

$$\begin{aligned} E[\log Y(d, z_1, \ldots , z_k)]-E[\log Y(0, z_1, \ldots , z_k)]= \beta _1 x_1 + \beta _2 x_2=\log (1-\text {BMR}). \end{aligned}$$

If a loss of BMR $\times 100\%$ happens before the $d_0$ then $\beta _1 d_0<\log (1-\text {BMR})$, and the BMD will be

16 $$\begin{aligned} \text {BMD}=\frac{\log (1-\text {BMR})}{\beta _1} \end{aligned}$$

If a loss of BMR$\times 100\%$ happens after the $d_0$ then $\beta _1 d_0>\log (1-\text {BMR})$, and if the slope after the break-point is negative ($\beta _2<0$), the BMD will be

17 $$\begin{aligned} \text {BMD}=d_0+\frac{\log (1-\text {BMR})-\beta _1 d_{0}}{\beta _2} \end{aligned}$$

The BMD is infinity if $\beta _1 d_0> \log (1-\text {BMR})$ and $\beta _2>0$.

In applications, the BMD is estimated by first estimating model (15) and then plugging regression coefficients into expression (16) or (17). The BMDL, the lower confidence limit of the BMD, can be determined by first calculating the lower confidence limit for $E[\log Y(d, z_1, \ldots , z_k)]-E[\log Y(0,z_1, \ldots , z_k)]=\beta _1 x_1 + \beta _2 x_2$ as a function of *d*, and then determining when this function reaches $\log (1-\text {BMR})$.

Below $d_0$, the lower limit for $E[\log Y(d, z_1, \ldots , z_k)]-E[\log Y(0,z_1, \ldots , z_k)]$ is $lowercl(\beta _1) \, d$, where $lowercl(\beta _1)$ is a lower 95% confidence limit of $\beta _1$. Here this value is given by $lowercl(\beta _1)=\widehat{\beta }_1 -t(df,95) se_1$, where $se_1$ is the standard error of the regression coefficient $\widehat{\beta }_1$ and *t*(*df*, 95) is the 95’th percentile of the *t*-distribution with degrees of freedom given by $df=n-k$, where *k* is the number of parameters in the model for the mean. So if $(\widehat{\beta }_1 - t(df,95) se_1) d_0 < \log (1-\text {BMR})$ then we get

18 $$\begin{aligned} \text {BMDL}=\frac{\log (1-\text {BMR})}{\widehat{\beta }_1 - t(df,95) se_1} \end{aligned}$$

For $d>d_0$ the lower confidence limit for $E[\log Y(d,z_1, \ldots , z_k)]-E[\log Y(0,z_1, \ldots , z_k)]$ can be derived as

$$\begin{aligned} \beta _1 d_0 + \beta _2 x_2- t(df,95) \sqrt{v_1 d_0^2 + v_2 x_ 2^2+2 \, cov \, d_0 \, x_2}, \end{aligned}$$

where $v_1,v_2$ and *cov* are the variances of $\widehat{\beta }_1, \widehat{\beta }_2$ and the covariance between these estimators. The BMDL can be found as the value of *d* where the confidence limit is $\log (1-\text {BMR})$. The solution can be found by solving a 2nd degree polynomial equation ($a x^2 + b x + c=0$) with $a=t(df,95)^2 v_2 -\beta _2^2$, $b=2[t(df,95)^2 \, d_0 \,cov+ \beta _2 \log (1-\text {BMR}) - \beta _1 \beta _2 d_0]$ and $c=t(df,95)^2 v_1 d_0^2-[\log (1-\text {BMR})-\beta _1 d_0]^2$ to get the level of $x_2$. Then the BMDL is given by the sum of $d_0$ and the solution for $x_2$, but as there will be two solutions one must chose the one where the sum is larger than $d_0$ and lower than the estimated value of the BMD.
